# Supplementary material for: Oncogene-induced senescence in hematopoietic progenitors features myeloid restricted hematopoiesis, chronic inflammation and histiocytosis
Source: Nat Commun. 2021 Jul 27;12:4559. doi: 10.1038/s41467-021-24876-1 (PMC8316479; doi:10.1038/s41467-021-24876-1)
Supplement: Supplementary file 1 — Supplementary Information [file 41467_2021_24876_MOESM1_ESM.pdf]

# Oncogene-induced senescence in hematopoietic progenitors features myeloid restricted hematopoiesis, chronic inflammation and histiocytosis

Riccardo Biavasco, Emanuele Lettera, Kety Giannetti, Diego Gilioli, Stefano Beretta, Anastasia Conti, Serena Scala, Daniela Cesana, Pierangela Gallina, Margherita Norelli, Luca Basso-Ricci, Attilio Bondanza, Giulio Cavalli, Ponzoni Maurilio, Lorenzo Dagna, Claudio Doglioni, Aiuti Alessandro, Ivan Merelli, Raffaella Di Micco and Eugenio Montini.

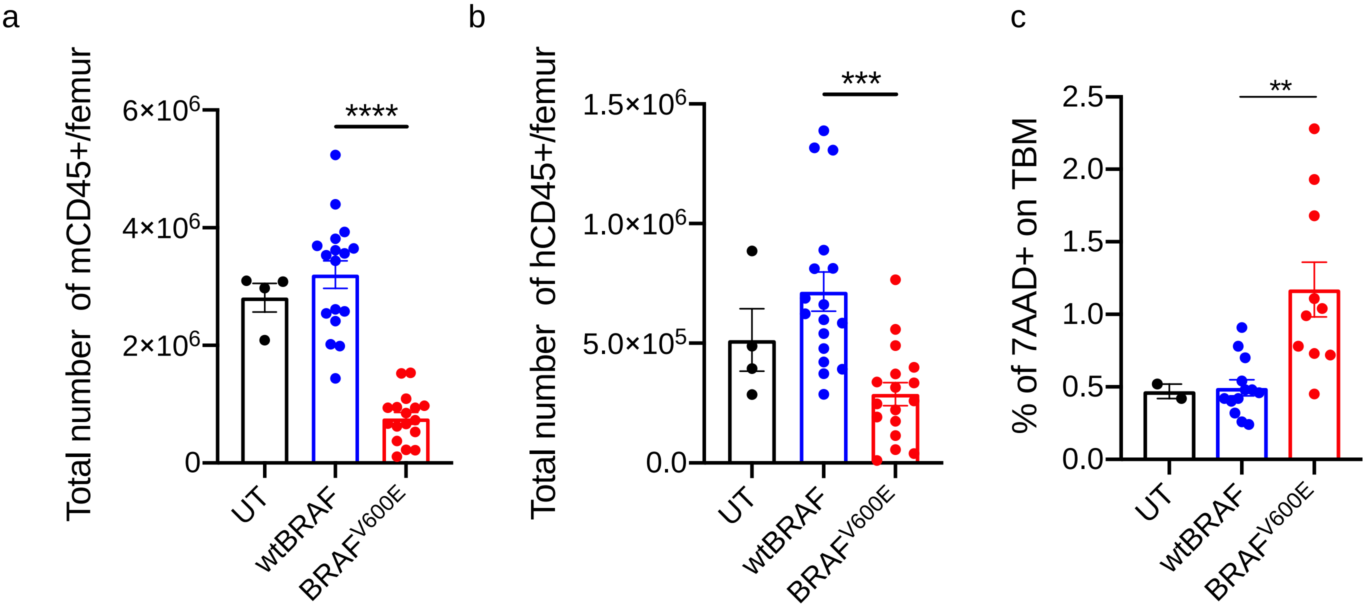


d

| Mouse ID | Vector | Spleen | Liver | Lung | Kidney | Heart | Thymus | Brain | Meninges | Gut | BM femur | BM sternum |
|---|---|---|---|---|---|---|---|---|---|---|---|---|
| RH1655A | GFP | 0 | 0 | 0 | 0 | 0 | 0 | 0 | 0 | 0 | 0 | 0 |
| RH1655B | | 0 | 0 | 0 | 0 | 0 | 0 | 0 | 0 | 0 | 0 | 0 |
| RH1655C | | 0 | 0 | 0 | 0 | 0 | 0 | 0 | 0 | 0 | 0 | 0 |
| RH1656C | UT | 0 | 0 | 0 | 0 | 0 | 0 | 0 | 0 | 0 | 0 | 0 |
| RH1656D | | 0 | 0 | 0 | 0 | 0 | 0 | 0 | 0 | 0 | 0 | 0 |
| RH1656E | wtBRAF | 0 | 0 | 0 | 0 | 0 | 0 | 0 | 0 | 0 | 0 | 0 |
| RH1656F | | 0 | 0 | 0 | 0 | 0 | 0 | 0 | 0 | 0 | 0 | 0 |
| RH1656A | | 0 | 0 | 0 | 0 | 0 | 0 | 0 | 0 | 0 | 0 | 0 |
| RH1656B | | 0 | 0 | 0 | 0 | 0 | 0 | 0 | 0 | 0 | 0 | 0 |
| RH1655D | $BRAF^{V600E}$ | 1 | 0 | 1 | 1 | 0 | NA | 0 | 1+ | 0 | 3 | 3+ |
| RH1655E | | NA | 1 | 2 | 1+ | 0 | NA | 0 | 1+ | 0 | 3 | 3+ |
| RH1655F | | 0 | 0 | 0 | 0 | 0 | 0 | 0 | 0 | 0 | 2+/3+ | 2+/3+ |

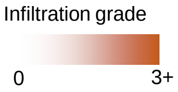


Supplementary Figure 1. Bone marrow cellularity and infiltration grade of organs in transplanted mice at euthanasia. a, b) Total number of murine (a) and human (b) $CD45^{+}$ ($mCD45^{+}$ and hCD45 respectively) cells *per* femur from mice wtBRAF (n=17 animals), $BRAF^{V600E}$ (n=17 animals) and UT (n=4 animals) groups (euthanized at 24 days after transplant). Data are presented as mean value+/-SEM. Statistical test: Kruskal-Wallis with Dunn's multiple comparisons ****p<0.0001, ***p<0.001. c) Relative percent of necrotic cells ($7AAD^{+}$) on total bone marrow of mice transplanted from wtBRAF (n=13 animals), $BRAF^{V600E}$ (n=10 animals) and UT (n=2 animals) groups (euthanized at 24 days after transplant). Data are presented as mean value+/-SEM. Statistical test: Kruskal-Wallis with Dunn's multiple comparisons. **p<0.005 (p=0.0027). d) The indicated organs were collected at euthanasia from mice transplanted with HSPCs transduced with a vector expressing GFP (GFP), or wild type BRAF (wtBRAF), mutated $BRAF^{V600E}$ or untransduced cells (UT). Two independent pathologists evaluated the infiltration grade and provided the score. All graphs in this figure display data from UT group in black, wtBRAF in blue and from $BRAF^{V600E}$ in red.

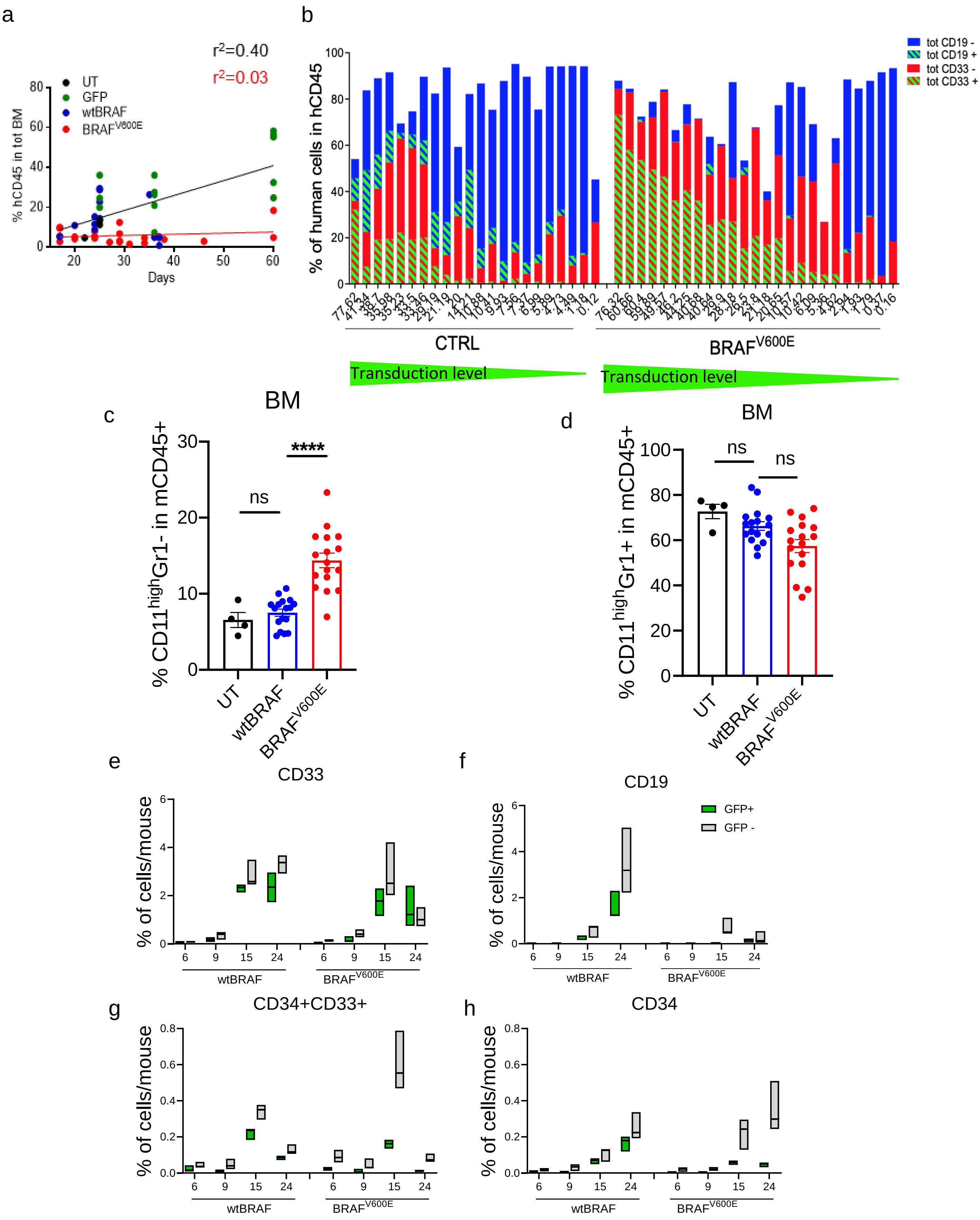
a
r²=0.40
r²=0.03
UT
GFP
wtBRAF
BRAFV600E
% hCD45 in tot BM
Days
b
% of human cells in hCD45
tot CD19 -
tot CD19 +
tot CD33 -
tot CD33 +
CTRL
BRAFV600E
Transduction level
Transduction level
c
BM
% CD11highGr1- in mCD45+
ns
****
UT
wtBRAF
BRAFV600E
d
BM
% CD11highGr1+ in mCD45+
ns
ns
UT
wtBRAF
BRAFV600E
e
CD33
% of cells/mouse
wtBRAF
BRAFV600E
f
CD19
GFP+
GFP -
% of cells/mouse
wtBRAF
BRAFV600E
g
CD34+CD33+
% of cells/mouse
wtBRAF
BRAFV600E
h
CD34
% of cells/mouse
wtBRAF
BRAFV600E

Supplementary Figure 2. Impact of oncogene activation on murine and human HSPC subpopulations. a) Engraftment levels of human $CD45^+$ cells in bone marrow (BM) of individual mice at the time of euthanasia shown in Figure 2b. Trendlines of the engraftment levels for controls (black) and mice from the $BRAF^{V600E}$ group (red) are shown. Goodness of fit measured as $R^2$. b) Percentage of $GFP^+$ and GFP- myeloid ($CD33^+$) and B ($CD19^+$) cells within $hCD45^+$ cells at time of euthanasia. Values on x-axis were ordered by transduction level. c, d) percentage of c) mouse monocytic/macrophagic lineage cells (CD11+high/$Gr1^-$/$mCD45^+$) or d) mouse granulocytes ($CD11^+$high/$Gr1^+$/$mCD45^+$) from wtBRAF (blue, n=17 mice) and $BRAF^{V600E}$ (red, n=17 mice) group compared to control groups (UT, black, n=4 mice). Data are presented as mean value+/-SEM. Statistical test: Kruskal-Wallis with Dunn's multiple comparisons. ****p<0.0001, ns: not significant. e-h) Relative percentage of $GFP^+$ (green plots) and $GFP^-$ (grey plots) human cells (CD33, CD19, $CD34^+$/$CD33^+$, $CD34^+$) on total BM at the indicated time points from wtBRAF and $BRAF^{V600E}$ transplantation groups. n=3 mice for each time point.

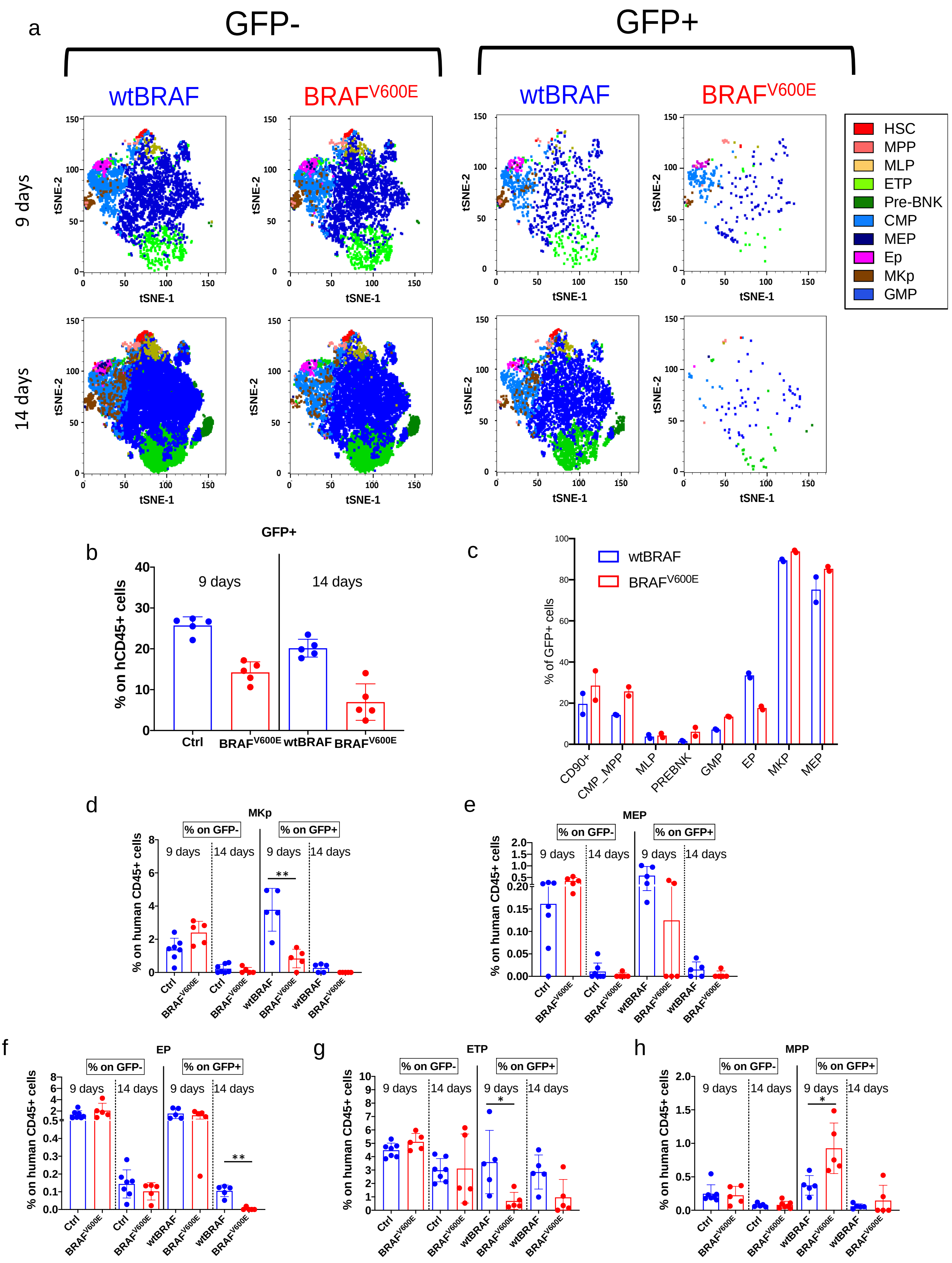
a
GFP-
GFP+
wtBRAF
BRAF V600E
9 days
14 days
tSNE-1
tSNE-2
HSC
MPP
MLP
ETP
Pre-BNK
CMP
MEP
Ep
MKp
GMP
b
GFP+
% on hCD45+ cells
9 days
14 days
Ctrl
BRAF V600E
wtBRAF
c
% of GFP+ cells
CD90+
CMP_MPP
MLP
PREBNK
GMP
EP
MKP
MEP
d
MKp
% on GFP-
% on GFP+
% on human CD45+ cells
e
MEP
f
EP
g
ETP
h
MPP

Supplementary Figure 3. Impact of oncogene activation on HSPC subpopulations frequency. a) tSNE analysis based on the surface expression of the whole blood dissection (WBD) HSPC markers. Cells were colored according to the gating strategy definition. All human CD45 events of both wtBRAF and $BRAF^{V600E}$ experimental groups were analyzed together and then stratified based on GFP expression. The composition of HSPCs is similar in all the samples analyzed; however, mice transplanted with $BRAF^{V600E}$-expressing HSPCs show a progressive loss of most HSPC subsets. b) Levels of transduction (percent of $GFP^+$ within $hCD45^+$) in mice from the wtBRAF (blue) and $BRAF^{V600E}$ (red) groups relative to Figure 2k-p, (n=5 for each time point). Data are presented as mean value+/-SD. c) Levels of transduction (percent of $GFP^+$ cells) in different HSPC subsets (n=2 independent donors) after 120h in vitro post-transduction. d-h) Percentage of human HSPC subpopulations within $GFP^-$ and $GFP^+$ fractions in BM cells isolated from transplanted mice, $BRAF^{V600E}$ group in red, CTRL group: cumulative values of mice transplanted with untransduced or wtBRAF-expressing HSPCs, in blue. Mice were euthanized at 9 and 14 days after HSPC transplant. (HSC-enriched fraction=$CD90^+$; CMP=Common-Myeloid Progenitors; MPP=Multi-potent progenitors; MLP=multi-lymphoid progenitors; PreBNK=B and NK cell progenitors; GMP=Granulocyte/Monocyte Progenitors; EP=Erythroid progenitors; MKP=Megakaryocyte Progenitors; MEP=Megakaryocyte/Erythrocyte Progenitors; ETP=Early T cell progenitors). n=5 for each time point. Data are presented as mean value+/-SD. Statistical test: Two-tailed Mann-Whitney. $*p<0.05$, $**p<0.01$.

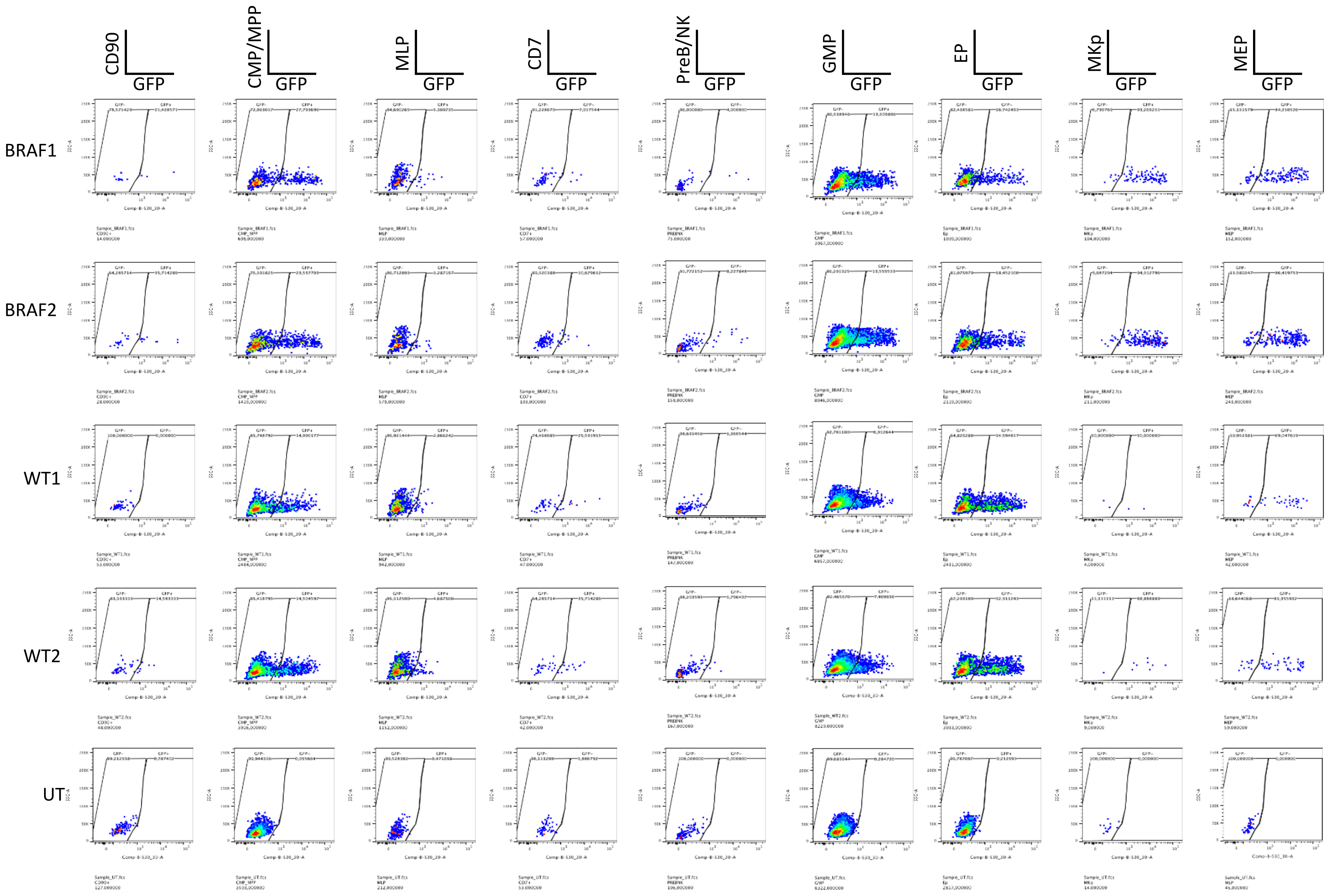

CD90
CMP/MPP
MLP
CD7
PreB/NK
GMP
EP
MKp
MEP
GFP
BRAF1
BRAF2
WT1
WT2
UT

Supplementary Figure 4 Representative FACS plots of the different HSPC subsets analyzed. Fluorophores are indicated in the axis legend, populations marked are shown on the scheme on top of each column. The gating strategy represented in this figure corresponds to FACS data panels in Figure 2k-p and Supplementary Figure 3c-h

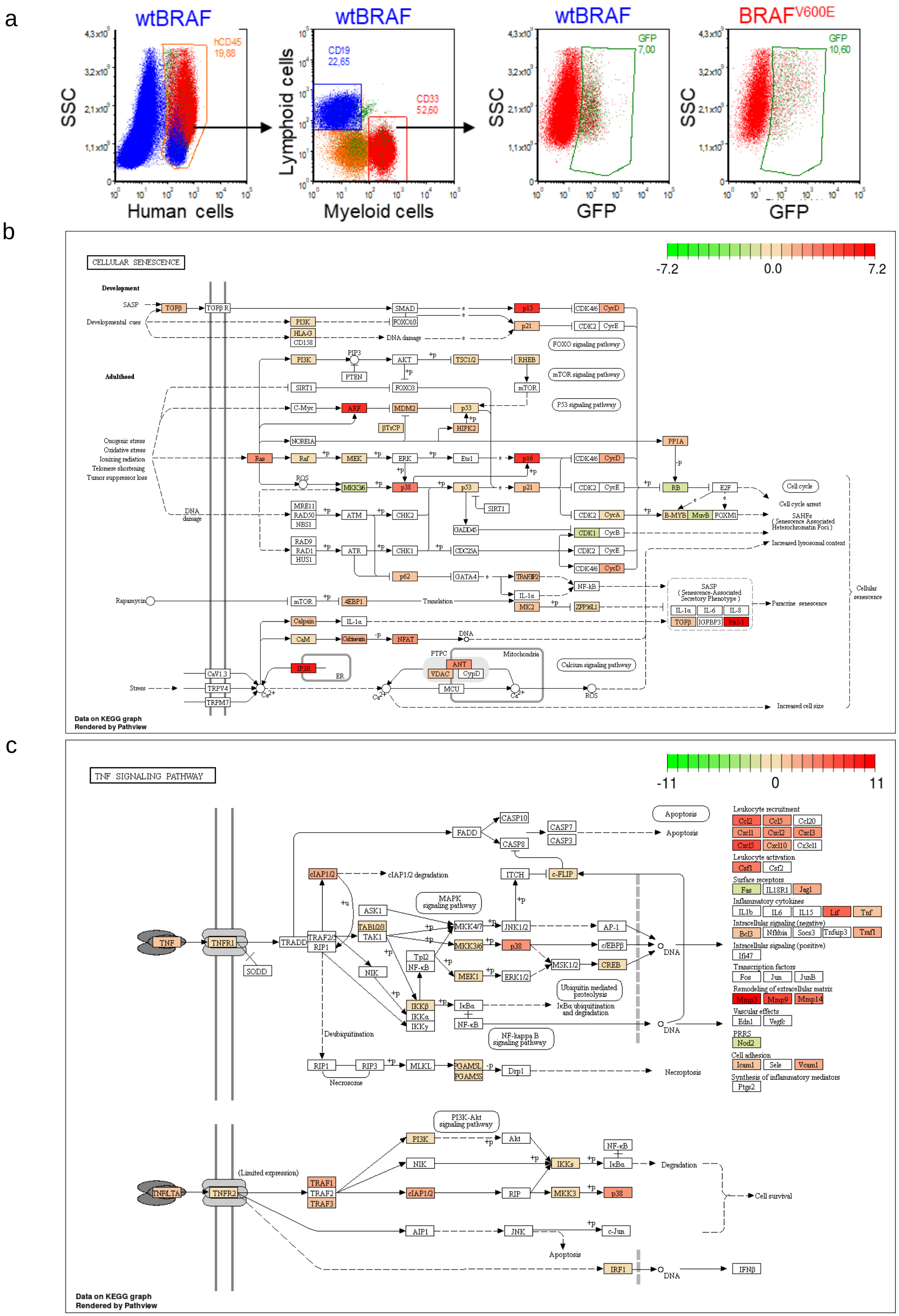

a
wtBRAF
wtBRAF
wtBRAF
BRAFV600E
SSC
Human cells
hCD45
19,88
Lymphoid cells
Myeloid cells
CD19
22,65
CD33
52,60
GFP
GFP
7,00
GFP
10,60
b
CELLULAR SENESCENCE
-7.2
0.0
7.2
Development
Adulthood
SASP
Developmental cues
Oncogenic stress
Oxidative stress
Ionizing radiation
Telomere shortening
Tumor suppressor loss
DNA damage
Rapamycin
Stress
FOXO signaling pathway
mTOR signaling pathway
P53 signaling pathway
Calcium signaling pathway
Cell cycle
Cell cycle arrest
SAHFs
( Senescence Associated Heterochromatin Foci )
Increased lysosomal content
SASP
( Senescence-Associated Secretory Phenotype )
Paracrine senescence
Cellular senescence
Increased cell size
Translation
Mitochondria
PTPC
ER
Data on KEGG graph
Rendered by Pathview
c
TNF SIGNALING PATHWAY
-11
0
11
Apoptosis
Leukocyte recruitment
Leukocyte activation
Surface receptors
Inflammatory cytokines
Intracellular signaling (negative)
Intracellular signaling (positive)
Transcription factors
Remodeling of extracellular matrix
Vascular effects
PRRS
Cell adhesion
Synthesis of inflammatory mediators
MAPK signaling pathway
cIAP1/2 degradation
Ubiquitin mediated proteolysis
IκBα ubiquitination and degradation
NF-kappa B signaling pathway
Deubiquitination
Necrosome
Necroptosis
PI3K-Akt signaling pathway
(Limited expression)
Degradation
Cell survival
DNA
Data on KEGG graph
Rendered by Pathview

Supplementary Figure 5. Global transcriptomic analyses of oncogene-expressing myeloid cells. a) Sorting strategy for $CD33^{+}/GFP^{+}$ and $CD33^{+}$/GFP- cells for RNA-sequencing analysis. b, c) Schematic representation of the senescence and TNF signaling pathways (using KEGG Graph data) showing Log2FC expression of DEGs from $BRAF^{V600E}$ group compared to controls.

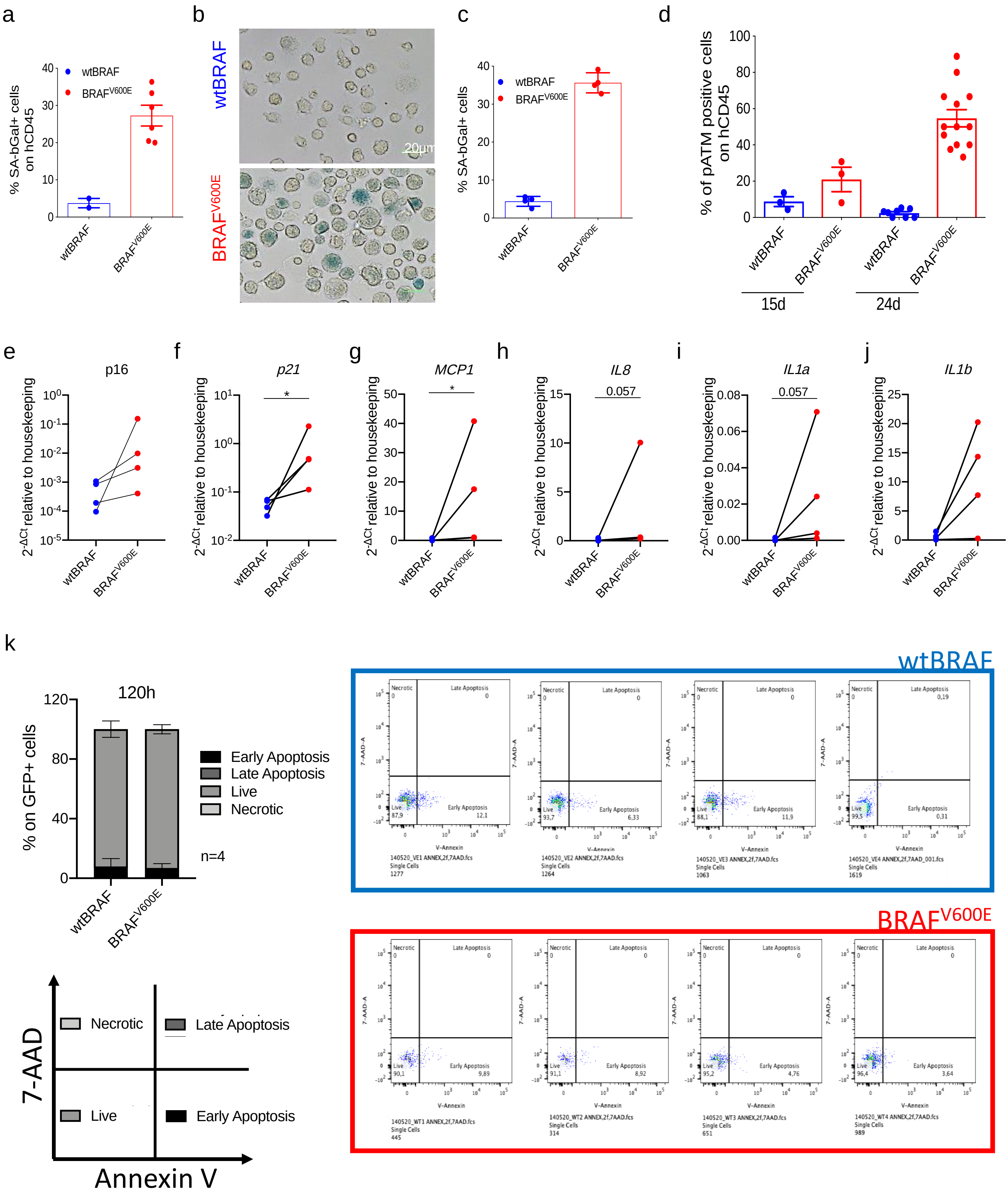
a
% SA-bGal+ cells on hCD45
wtBRAF
BRAFV600E
b
wtBRAF
BRAFV600E
20µm
c
% SA-bGal+ cells
wtBRAF
BRAFV600E
d
% of pATM positive cells on hCD45
wtBRAF
BRAFV600E
15d
24d
e
p16
f
p21
*
g
MCP1
*
h
IL8
0.057
i
IL1a
0.057
j
IL1b
2-ΔCt relative to housekeeping
k
120h
% on GFP+ cells
Early Apoptosis
Late Apoptosis
Live
Necrotic
n=4
wtBRAF
BRAFV600E
Necrotic
Late Apoptosis
Live
Early Apoptosis
7-AAD
Annexin V
7-AAD-A
V-Annexin
140520_VE1 ANNEX,2f,7AAD.fcs
140520_VE2 ANNEX,2f,7AAD.fcs
140520_VE3 ANNEX,2f,7AAD.fcs
140520_VE4 ANNEX,2f,7AAD_001.fcs
140520_WT1 ANNEX,2f,7AAD.fcs
140520_WT2 ANNEX,2f,7AAD.fcs
140520_WT3 ANNEX,2f,7AAD.fcs
140520_WT4 ANNEX,2f,7AAD.fcs
Single Cells

Supplementary Figure 6. $BRAF^{V600E}$ expression in HSPCs promotes oncogene-induced senescence. a) Quantification of SA-β-gal positive cells within human $CD45^+$ cells isolated from BM of mice transplanted with wtBRAF or $BRAF^{V600E}$ expressing HSPCs at 24 days post transplantation. Each dot represents an independent measurement from a pool of 3 mice for wtBRAF and $BRAF^{V600E}$ groups. Lines indicate mean values±SEM. b) Representative images of SA-β-gal positive cells in wtBRAF and $BRAF^{V600E}$-expressing HSPCs 8 days after transduction in liquid culture. Scale bar=20µm. c) Quantification of SA-β-gal positive cells from (b). Each dot represent and independent donor. d) Percentage of pATM (S1981) positive cells within the human graft ($hCD45^+$) in the BM of mice transplanted with wtBRAF and $BRAF^{V600E}$- expressing cells at the indicated time points post-transplantation. Each dot represents independent measurement from a pool of 3 mice for wtBRAF and $BRAF^{V600E}$ groups. Lines indicate mean values±SEM. e-j) Relative mRNA expression of cell cycle inhibitors p21 (E, p=0.0286) and p16 (f) and SASP cytokines MCP1 (g, p=0.0286), IL8 (h), IL1α (i), IL1β (j), in wtBRAF and $BRAF^{V600E}$-expressing cells as determined by RT-qPCR at 5 days post-transduction. Gene expression data are represented as $2^{\wedge-\Delta Ct}$ relative to housekeeping gene. Each dot represents an independent donor transduced with wtBRAF (N=4) or $BRAF^{V600E}$ (N=4). The connecting lines between dots indicate same donor origin. Statistical test: Two-tailed Mann-Whitney. *$p<0.05$. k) Percentage of alive, apoptotic and necrotic cells in *in vitro* cultured HSPCs 120h post transduction with wtBRAF or $BRAF^{V600E}$ expressing vectors; the scheme on the bottom left illustrates the gating strategy, the frames on the right (blue for wtBRAF and red for $BRAF^{V600E}$) contain representative FACS plots. Data are presented as mean value+/-SD. All graphs in this figure display data from wtBRAF in blue and from $BRAF^{V600E}$ in red.

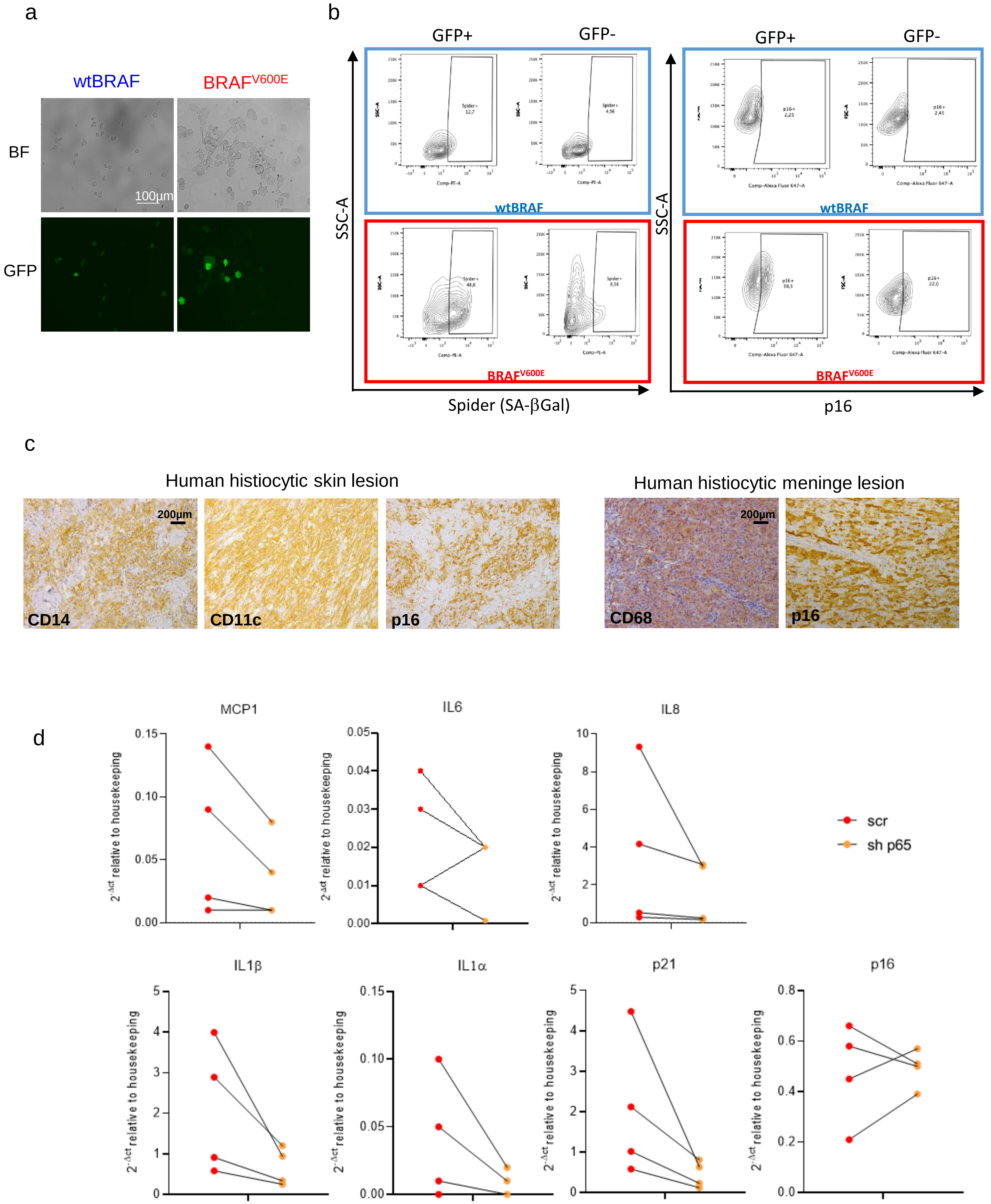

a
wtBRAF
BRAFV600E
BF
GFP
100µm
b
GFP+
GFP-
wtBRAF
BRAFV600E
SSC-A
Spider (SA-βGal)
p16
c
Human histiocytic skin lesion
Human histiocytic meninge lesion
200µm
CD14
CD11c
p16
CD68
d
MCP1
IL6
IL8
IL1β
IL1α
p21
2-Δct relative to housekeeping
scr
sh p65

Supplementary Figure 7. $BRAF^{V600E}$ expression in HSPCs promotes oncogene-induced senescence through RELA/p65 mediated expression of cytokines. a) Morphological changes in $BRAF^{V600E}$- expressing ($GFP^{+}$) and bystander (GFP-) HSPCs compared to control at 9 days post-transduction. BF=Brightfield, GFP indicates transduced cells. Scale bar=100µm. b) Representative FACS plots of the data summarized in Figure 5c, d. c) Immunohistochemical characterization of skin and meninges lesions for expression of the cell cycle inhibitor p16 and myeloid/macrophage markers (CD11c, CD14 and CD68). Scale bar: 200 µm. d) Expression levels of SASP cytokines in $BRAF^{V600E}$-expressing cells upon treatment with a scrambled sequence shRNA control (scr) or shRNA against RELA/p65. Relative mRNA expression was determined by RT-qPCR at 5 days post-transduction and are represented as $2^{\wedge-\Delta Ct}$ relative to housekeeping gene. Each dot represents an independent donor transduced with $BRAF^{V600E}$ vector (N=4). The connecting lines between dots indicate same donor origin.

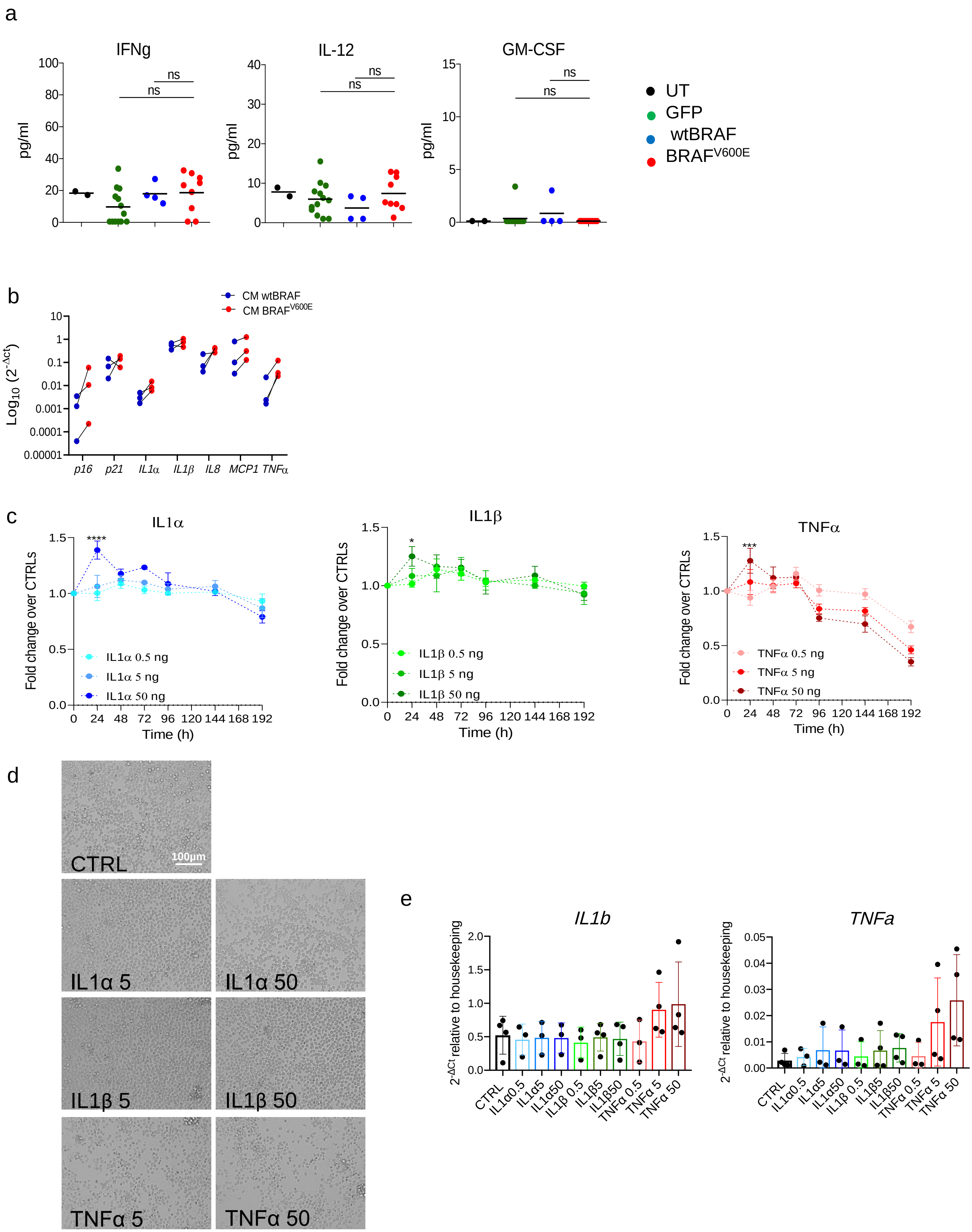

a
IFNg
IL-12
GM-CSF
pg/ml
ns
UT
GFP
wtBRAF
BRAFV600E
b
CM wtBRAF
CM BRAFV600E
Log10 (2-Δct)
p16 p21 IL1α IL1β IL8 MCP1 TNFα
c
IL1α
IL1β
TNFα
Fold change over CTRLs
IL1α 0.5 ng
IL1α 5 ng
IL1α 50 ng
IL1β 0.5 ng
IL1β 5 ng
IL1β 50 ng
TNFα 0.5 ng
TNFα 5 ng
TNFα 50 ng
Time (h)
d
CTRL
100µm
IL1α 5
IL1α 50
IL1β 5
IL1β 50
TNFα 5
TNFα 50
e
IL1b
TNFa
2-ΔCt relative to housekeeping
CTRL IL1α0.5 IL1α5 IL1α50 IL1β 0.5 IL1β5 IL1β50 TNFα 0.5 TNFα 5 TNFα 50

Supplementary Figure 8. Impact of SASP factors on HSPC biology. a) Concentrations of the indicated human pro-inflammatory cytokines in mice transplanted with untransduced (UT, n=2 animals) HSPCs or HSPCs transduced with GFP (n=13), wtBRAF (n=4) or $BRAF^{V600E}$ (n=13). Lines represent means. Each dot represents an individual mouse. Statistical test: Kruskal-Wallis with Dunn's multiple comparisons. ns=non significant. b) Gene expression analysis of senescence-associated markers in CM-treated HSPCs. Data are represented as $2^{\wedge-\Delta Ct}$ relative to housekeeping gene. Each dot represents an independent donor. The connecting lines between dots indicate same donor origin (n=3 independent donors). c) Dashed lines indicate the fold change in growth of cytokine (IL1α, IL1β or TNFα)-treated HSPCs over controls. This is a representation of the data shown in Figure 7f-h. Statistical analysis was performed on data in Figure 7f-h with linear mixed-effects model (LME), see also Source Data file. Technical replicates: CTRL(n=5), IL1α-IL1β-TNFα(n=4). CTRL values are repeated. Data are presented as mean values+/-SD. d) Representative images of IL1α, IL1β and TNFα treated HSPCs and untreated controls at 7 days post-treatment. Cytokine concentrations are indicated. Scale bar=100µm. e) Expression levels of IL1β and TNFα in IL1α, IL1β or TNFα-treated HSPCs and untreated controls at 7 days post-treatment. Dots indicate independent biological replicates (3 independent donors for groups: CTRL, IL1α0.5, IL1α5, IL1α50, IL1β0.5 and TNFα0.5; 4 independent donors for groups: IL1β5, IL1β50, TNFα5, TNFα50). Data are presented as mean+/-SD.

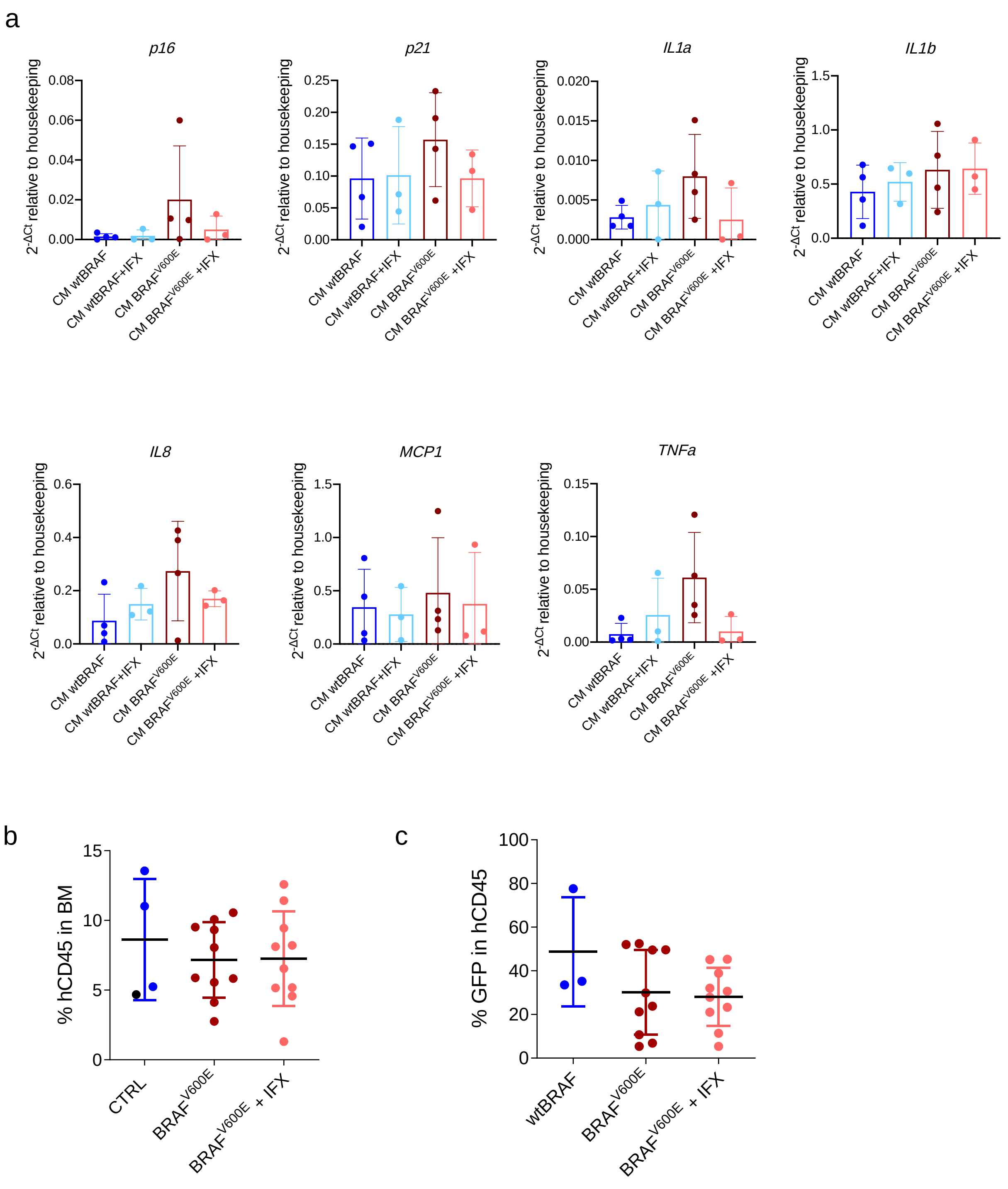
a
p16
p21
IL1a
IL1b
IL8
MCP1
TNFa
2-ΔCt relative to housekeeping
CM wtBRAF
CM wtBRAF+IFX
CM BRAFV600E
CM BRAFV600E +IFX
b
% hCD45 in BM
CTRL
BRAFV600E
BRAFV600E + IFX
c
% GFP in hCD45
wtBRAF
BRAFV600E
BRAFV600E + IFX

Supplementary Figure 9. Effects of TNFα inhibition *in vitro* and *in vivo*. a) Gene expression analysis of data in Figure 8c for senescence-associated markers in CM-treated HSPCs in presence or absence of infliximab (IFX). Data are represented as $2^{\wedge-\Delta Ct}$ relative to housekeeping gene and presented as mean value+/-SD. Each dot represents an independent donor. Untreated n=4; treated n=3 biological replicates. b-c) Percentage of (b) human cells ($hCD45^{+}$) or (c) $GFP^{+}$ cells in mice transplanted with CTRL (black dot indicates UT=untransduced, blue dots indicate wtBRAF-expressing HSPCs) or $BRAF^{V600E}$-expressing cells (in presence (pink) or absence (red) of IFX). (CTRL n=4, $BRAF^{V600E}$ n=10, $BRAF^{V600E}$+IFX n=10). Data are presented as mean value+/-SD.

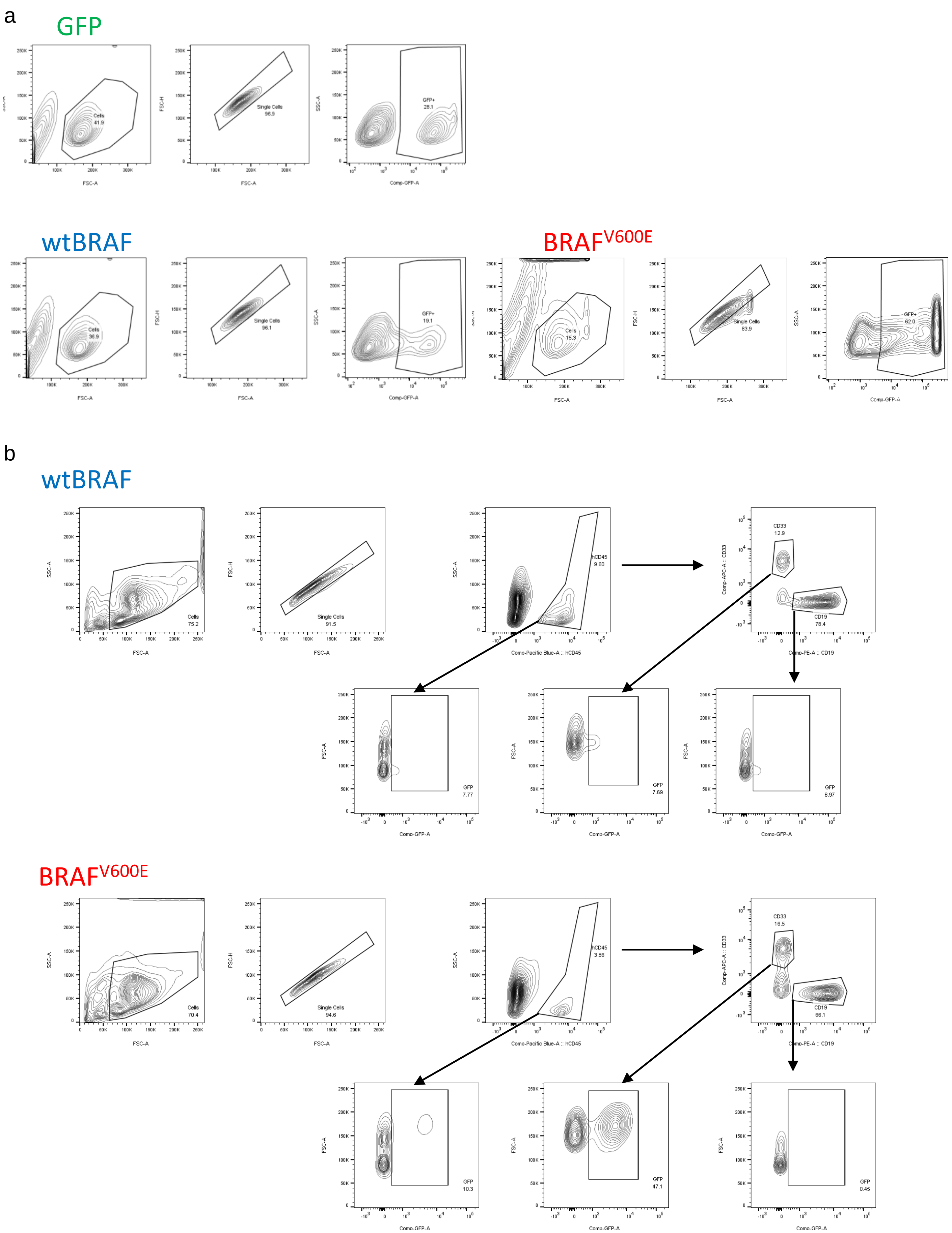
a
GFP
Cells
41.9
Single Cells
96.9
GFP+
28.1
FSC-A
FSC-H
SSC-A
Comp-GFP-A
wtBRAF
Cells
36.9
Single Cells
96.1
GFP+
19.1
BRAFV600E
Cells
15.3
Single Cells
83.9
GFP+
62.0
b
wtBRAF
Cells
75.2
Single Cells
91.5
hCD45
9.60
Comp-Pacific Blue-A :: hCD45
CD33
12.9
CD19
78.4
Comp-APC-A :: CD33
Comp-PE-A :: CD19
GFP
7.77
GFP
7.69
GFP
6.97
BRAFV600E
Cells
70.4
Single Cells
94.6
hCD45
3.86
CD33
16.5
CD19
66.1
GFP
10.3
GFP
47.1
GFP
0.45

Supplementary Figure 10 Flow cytometry gating strategies for evaluation of in vitro transduction and in vivo human hematopoiesis. (a) Representative plots for GFP (green), wtBRAF (blue) and $BRAF^{V600E}$ (red) transduced HSPCs. The gating strategy represented in this figure corresponds to data panels in Figure 1c and 6e. (b) Representative plots for human hematopoiesis analyses of BM retrivied from mice of the wtBRAF (blue) and $BRAF^{V600E}$ (red) groups. The gating strategy represented in this figure corresponds to data panels in figure 2a-h, 2j, 8f-g and Supplementary Figure 2a-b, 2e-h, 3b and 9b-c.

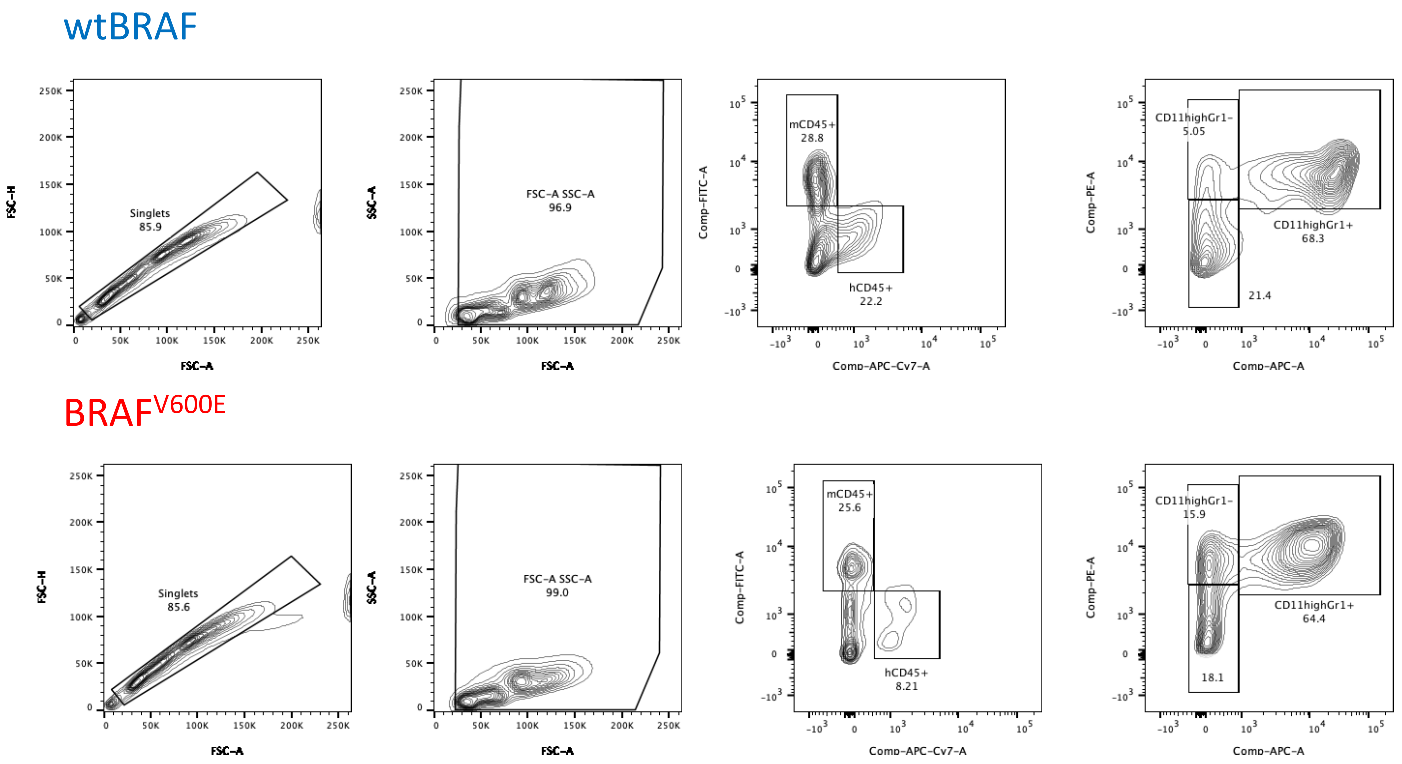


Supplementary Figure 11 Flow cytometry gating strategy for evaluation of murine hematopoiesis. Representative plots for wtBRAF (blue) and BRAF$^{V600E}$ (red) transduced HSPCs retrieved from BM of transplanted mice. The gating strategy represented in this figure corresponds to data panels in Supplementary Figure 2c-d.

**Supplementary Table 1. RT-qPCR Primer list**

| Target | Optimal concentration of primers | Sequence fw | Sequence rev |
|---|---|---|---|
| *CDKN1A* | 90 nM | CAGCATGACAGATTTCTA CCACTC | CTCGCGCTTCCAGGACTG |
| *CDKN2A* | 160 nM | CCAACGCACCGAATAGTT ACG | GCGCTGCCCATCATCATG |
| *IL1α* | 120 nM | GGTTGAGTTTAAGCCAAT CCA | TGCTGACCTAGGCTTGATGA |
| *IL1β* | 100 nM | GCTCAAGTGTCTGAAGC AGCC | CAGCTTCAAAGAACAAGTCATCCT |
| *IL6* | 130 nM | GATTCAATGAGGAGACTT GCCTGG | CTCACTACTCTCAAATCTGTTCTGG |
| *IL8* | 100 nM | CATCTCACTGTGTGTAAA CATGAC | CCTTGGCAAAACTGCACCTTCAC |
| *TNFα* | 100 nM | CCAGGGACCTCTCTCTAA TCAGC | GGTTTGCTACAACATGGGCTAC |
| *MCP1* | 150 nM | CTGTGATCTTCAAGACCA TTGTG | AGTTTGGGTTTGCTTGTCCAG |
| *GUSB* | 350 nM | CTGACACCTCCAAGTATC CCAAG | GTCGTGTACAGAAGTACAGACCGC |
