# Supplementary material for: Oncogene-induced senescence in hematopoietic progenitors features myeloid restricted hematopoiesis, chronic inflammation and histiocytosis
Source: Nat Commun. 2021 Jul 27;12:4559. doi: 10.1038/s41467-021-24876-1 (PMC8316479; doi:10.1038/s41467-021-24876-1)
Supplement: Supplementary file 3 — Description of Additional Supplementary Files [file 41467_2021_24876_MOESM3_ESM.pdf]

Description of Additional Supplementary Files

Supplementary Data 1: List of DEGs from RNA-seq analysis
