# Supplementary material for: Oncogene-induced senescence in hematopoietic progenitors features myeloid restricted hematopoiesis, chronic inflammation and histiocytosis
Source: Nat Commun. 2021 Jul 27;12:4559. doi: 10.1038/s41467-021-24876-1 (PMC8316479; doi:10.1038/s41467-021-24876-1)
Supplement: Supplementary file 5 — Reporting Summary [file 41467_2021_24876_MOESM5_ESM.pdf]

nature research

Corresponding author(s): Eugenio Montini and Raffaella Di Micco

Last updated by author(s): 2021 April 09

Reporting Summary

Nature Research wishes to improve the reproducibility of the work that we publish. This form provides structure for consistency and transparency in reporting. For further information on Nature Research policies, see our Editorial Policies and the Editorial Policy Checklist.

Statistics

For all statistical analyses, confirm that the following items are present in the figure legend, table legend, main text, or Methods section.

n/a	Confirmed	
☐	☒	The exact sample size (*n*) for each experimental group/condition, given as a discrete number and unit of measurement
☐	☒	A statement on whether measurements were taken from distinct samples or whether the same sample was measured repeatedly
☐	☒	The statistical test(s) used AND whether they are one- or two-sided<br>*Only common tests should be described solely by name; describe more complex techniques in the Methods section.*
☐	☒	A description of all covariates tested
☐	☒	A description of any assumptions or corrections, such as tests of normality and adjustment for multiple comparisons
☐	☒	A full description of the statistical parameters including central tendency (e.g. means) or other basic estimates (e.g. regression coefficient) AND variation (e.g. standard deviation) or associated estimates of uncertainty (e.g. confidence intervals)
☒	☐	For null hypothesis testing, the test statistic (e.g. *F*, *t*, *r*) with confidence intervals, effect sizes, degrees of freedom and *P* value noted<br>*Give P values as exact values whenever suitable.*
☒	☐	For Bayesian analysis, information on the choice of priors and Markov chain Monte Carlo settings
☒	☐	For hierarchical and complex designs, identification of the appropriate level for tests and full reporting of outcomes
X	~~☒~~	Estimates of effect sizes (e.g. Cohen's *d*, Pearson's *r*), indicating how they were calculated

Our web collection on statistics for biologists contains articles on many of the points above.

Software and code

Policy information about availability of computer code

Data collection	RNA was quantified with the Qubit 2.0 Fluorometer (ThermoFisher) and quality was assessed by Agilent 4200 TapeStation (Agilent Technologies). Sequencing data was obtained from NextSeq 500 (Illumina). The quality of the reads was determined using FastQC and low-quality sequences were trimmed using trimmomatic. Reads were then aligned to the human reference genome (GRCh38/hg38) using STAR (STAR v2.5.3), with standard input parameters, and gene counts were produced using Subread featureCounts, using Genecode v31 as gene annotation. Gene counts were produced using Subread featureCounts, using Genecode v31 as gene annotation. Transcript counts were processed using R (v3.4.1) and the R/ Bioconductor package edgeR (v3.20.9), normalizing for library size using trimmed mean of M-values, and correcting p-values using FDR. Gene Set Enrichment Analysis (GSEA) was performed considering different datasets (Gene Ontology, KEGG Pathway Database, Reactome Pathway Database, Molecular Signatures Database) using clusterProfiler (v 3.8.1, http://bioconductor.org/packages/release/bioc/html/ clusterProfiler.html) by pre-ranking genes according to Log2FC values. Volcano plots have been used to display RNA-seq results plotting the statistical significance (P value) versus the magnitude of change (fold change). Fluorescent images were acquired using Leica SP2 and Leica SP5 Confocal microscopes. Where indicated, quantification of DDR foci in immunofluorescence images was conducted using ImageJ64 (version 1.47). Statistical analyses were performed with GraphPad Prism 8.0 and R (version 3.5). Flow cytometry data was collected with FACS DIVA software (version 6.5). Bioplex manager software /version 6.1 was used for cytokine quantification. Growth curves were analyzed using linear mixed-effects models (LME, Pinheiro et al. 2018; nlme: Linear and Nonlinear Mixed Effects Models. R Package version 3.1-137, <URL:https://CRAN.R-project.org/package=nlme>). Graphical output was generated through Prism 8.3.0 (GraphPad software).
Data analysis	TSNE plots were generated through FlowJo software plug-in. FACS data was analyzed with FlowJo software Version 10.5.3 (BD Biosciences) and the analysis and graphical output was generated through Prism 8.3.0 (GraphPad software). TSNE plots were generated through FlowJo software plug-in.

For manuscripts utilizing custom algorithms or software that are central to the research but not yet described in published literature, software must be made available to editors and reviewers. We strongly encourage code deposition in a community repository (e.g. GitHub). See the Nature Research guidelines for submitting code & software for further information.

Data

Policy information about availability of data

All manuscripts must include a data availability statement. This statement should provide the following information, where applicable:

- Accession codes, unique identifiers, or web links for publicly available datasets
- A list of figures that have associated raw data
- A description of any restrictions on data availability

All relevant data are included in the manuscript and its supplementary information files (also provided as a Source Data file), or from the corresponding authors upon reasonable request. The RNA-seq data from this study have been deposited in the NCBI Gene Expression Omnibus (GEO) under accession number: GSE144058. All software applications used were free or commercially available. databases used in this study are: gene Ontology, KEGG Pathway , Reactome Pathway and Molecular Signatures.

Field-specific reporting

Please select the one below that is the best fit for your research. If you are not sure, read the appropriate sections before making your selection.

[x] Life sciences   [ ] Behavioural & social sciences   [ ] Ecological, evolutionary & environmental sciences

For a reference copy of the document with all sections, see nature.com/documents/nr-reporting-summary-flat.pdf

Life sciences study design

All studies must disclose on these points even when the disclosure is negative.

Sample size	Sample size for each experiments was specifically calculated and defined depending on power statistics.
Data exclusions	No data was excluded
Replication	Technical and biological replicates have been specified
Randomization	Mice were randomly assigned to the experimental groups
Blinding	Initial data acquisition was performed in blind. Successive analyses required the knowledge of the samples in treatment groups

Reporting for specific materials, systems and methods

We require information from authors about some types of materials, experimental systems and methods used in many studies. Here, indicate whether each material, system or method listed is relevant to your study. If you are not sure if a list item applies to your research, read the appropriate section before selecting a response.

Materials & experimental systems

n/a	Involved in the study	
[ ]	[x]	Antibodies
[ ]	[x]	Eukaryotic cell lines
[x]	[ ]	Palaeontology and archaeology
[ ]	[x]	Animals and other organisms
[ ]	[x]	Human research participants
[ ]	[x]	Clinical data
[ ]	[x]	Dual use research of concern

Methods

n/a	Involved in the study	
[x]	[ ]	ChIP-seq
[ ]	[x]	Flow cytometry
[x]	[ ]	MRI-based neuroimaging

Antibodies

Antibodies used	see next page
Validation	All the antibodies belong to commercial brands that ensure quality control management of their products.

-

Antibodies used for Immunohistochemistry

- VENTANA anti-BRAF V600E (VE1) Mouse Monoclonal Primary Antibody Code 790-5095 (Roche Tissue Diagnostics, Tucson, AZ, USA)
- CD14 Ventana- cell marque, catalog 760-4523, clone EPR3653, lot v0001577
- CD1a Ventana - cell marque, catalog 760-4525, clone EP3622, lot v0001495- CD11c
- CD11c Novocastra, catalog number NCL-L-CD11c-563, clone CD11
- CD68/PGM1 DAKO, catalog number M0876, clone PG-M1, lotto 20058607
- CD207 Novocastra, catalog number nil-langherin, clone 12d6
- S100 Ventana, catalog 7602523, polyclonal, lot g12056
- Ki67 Ventana, catalog number 790-4286, clone 30-9, lot g28551
- p16 Ventana, catalog number , catalog number 8054713, clone E6H4, lot f20810
- TNF alpha Mouse Monoclonal Antibody [Clone P/T2] code 7124-MSM2-P (Neobiotechnology, Union City CA, USA)
- anti-GFP Polyclonal Antibody (cat. N: A-11122, lot:1925070, Invitrogen).

Antibodies used for immunofluorescence:

- Purified anti-CDKN2A (p16) Antibody, clone 15C10C30 (cat. N: 675602, Biolegend)
- Rabbit anti-53BP1 Antibody, Affinity Purified (cat. N: A300-272A, Bethyl Laboratories)
- Purified anti-ATM Phospho (Ser1981) Antibody, clone 10H11.E12 (cat. N: 651202, BioLegend)
- Donkey anti-Mouse IgG (H+L) Highly Cross-Adsorbed Secondary Antibody, Alexa Fluor 647 (cat. N: A-31571, Invitrogen)
- Donkey anti-Rabbit IgG (H+L) Highly Cross-Adsorbed Secondary Antibody, Alexa Fluor 568 (cat. N: A10042, Invitrogen)

Antibodies used for FACS:

- PE anti-human CD3 Antibody, clone: HIT3a (cat. N: 300308, Biolegend)
- BV605 anti-human CD3 Antibody, clone: OKT3 (cat. N: 317322, Biolegend)
- PE/Cyanine5 anti-human CD56 Antibody, clone 5.1H11 (cat. N: 362516, Biolegend)
- APC anti-human CD14 Antibody, clone: 63D3 (cat. N: 367118, Biolegend)
- PE/Cyanine7 anti-human CD41/61 Antibody, clone A2A9/6 (cat. N: 359812, Biolegend)
- PE/Cyanine7 anti-human CD13 Antibody, clone:WM15 (cat. N: 30172, Biolegend)
- APC anti-human CD13 Antibody, clone:WM15 (cat. N: 301706, Biolegend)
- APC/Cyanine7 anti-human CD34 Antibody, clone: 561 (cat. N: 343614, Biolegend)
- BV421 anti-human CD34 Antibody, clone: 561 (cat. N: 343610, Biolegend)
- APC/Cyanine7 anti-human CD45RA Antibody, clone HI100 (cat. N: 304128, Biolegend)
- PE anti-human CD33 Antibody, clone: WM53 (cat. N: 303404, Biolegend)
- APC anti-human CD33 Antibody, clone: WM53 (cat. N: 303408, Biolegend)
- PE anti-human CD66b Antibody, clone: G10F5 (cat. N: 305106, Biolegend)
- BUV737 anti-human CD38 Antibody, clone: HB7 (cat. N: 612824, BD Biosciences)
- Pacific Blue™ anti-human CD45 Antibody, clone:HI30 (cat. N: 304029, Biolegend)
- Brilliant Violet 510™ anti-human CD45 Antibody, clone:HI30 (cat. N: 304036, Biolegend)
- BUV395 anti-human CD45 Antibody, clone: HI30 (cat. N: 563792, BD Biosciences)
- FITC anti-mouse CD45 Antibody, clone: 30-F11 (cat. N: 103108, Biolegend)
- APC anti-human CD90 Antibody, clone: 5E10 (cat. N: 559869, BD Biosciences)
- BV786 anti-human CD10 Antibody, clone: HI10a (cat. N: 564960, BD Biosciences)
- BV650 anti-human CD11c Antibody, clone: B-ly6 (cat. N: 563404, BD Biosciences)
- PE anti-mouse/human CD11b Antibody, clone M1/70 (cat. N: 101207, Biolegend)
- PE anti-human CD19 Antibody, clone HIB19 (cat. N: 302208, Biolegend)
- APC anti-human CD19 Antibody, clone 4G7 (cat. N: 392504, Biolegend)
- APC/Cyanine7 anti-human CD19 Antibody, clone: SJ25C1 (cat. N: 363010, Biolegend)
- APC-R700 anti-human CD19 Antibody, clone: SJ25C1 (cat. N: 659121, BD Biosciences)
- BB700 anti-human CD7 Antibody, clone: M-T701 (cat. N: 566488, BD Biosciences)
- BV711 anti-human CD71 Antibody, clone: M-A712 (cat. N: 563767, BD Biosciences)
- APC anti-mouse Ly-6G/Ly-6C (Gr-1) Antibody, clone: RB6-8C5 (cat. N: 108411, Biolegend)
- Purified anti-CDKN2A (p16) Antibody, clone 15C10C30 (cat. N: 675602, Biolegend)
- Donkey anti-Mouse IgG (H+L) Highly Cross-Adsorbed Secondary Antibody, Alexa Fluor 647 (cat. N: A-31571, Invitrogen)

Eukaryotic cell lines

Policy information about cell lines

Cell line source(s)	NA
Authentication	NA
Mycoplasma contamination	NA
Commonly misidentified lines (See ICLAC register)	NA

Palaeontology and Archaeology

Specimen provenance	NA
Specimen deposition	NA
Dating methods	NA

☐ Tick this box to confirm that the raw and calibrated dates are available in the paper or in Supplementary Information.

Ethics oversight	NA

Note that full information on the approval of the study protocol must also be provided in the manuscript.

Animals and other organisms

Policy information about studies involving animals; ARRIVE guidelines recommended for reporting animal research

Laboratory animals	NOD.Cg-Prkdcscid IL2r tm1Wjl/SzJ mice were bred and kept in a dedicated pathogen-free animal facility, and were euthanized when showed signs of sickness. All animal handling procedures and light and dark cycles are described in the specific protocol IACUC #1131
Wild animals	No wild animals were used in this study
Field-collected samples	No field collected samples were used in this study
Ethics oversight	Mouse studies were performed according to protocols approved by the Animal Care and Use Committee of the San Raffaele Institute and communicated to the Ministry of Health and local authorities according to Italian law(IACUC #1131) .

Note that full information on the approval of the study protocol must also be provided in the manuscript.

Human research participants

Policy information about studies involving human research participants

Population characteristics	CD34+ HSPCs were freshly purified from human cord blood from healthy donors or purchased from Lonza. Mixes of cells from different donors were used in transplantation experiments
Recruitment	Each donor was tested and found non-reactive for the presence of HIV-I and hepatitis B virus.
Ethics oversight	CD34 were obtained upon informed consent from donors and upon approval by the Ospedale San Raffaele Bioethical Committee (TIGET09)

Note that full information on the approval of the study protocol must also be provided in the manuscript.

Clinical data

Policy information about clinical studies

All manuscripts should comply with the ICMJEguidelines for publication of clinical research and a completedCONSORT checklist must be included with all submissions.

Clinical trial registration	NA
Study protocol	NA
Data collection	NA
Outcomes	NA

Dual use research of concern

Policy information about dual use research of concern

Hazards

Could the accidental, deliberate or reckless misuse of agents or technologies generated in the work, or the application of information presented in the manuscript, pose a threat to:

No	Yes	
☒	☐	Public health
☒	☐	National security
☒	☐	Crops and/or livestock
☒	☐	Ecosystems
☒	☐	Any other significant area

Experiments of concern

Does the work involve any of these experiments of concern:

No	Yes	
☒	☐	Demonstrate how to render a vaccine ineffective
☒	☐	Confer resistance to therapeutically useful antibiotics or antiviral agents
☒	☐	Enhance the virulence of a pathogen or render a nonpathogen virulent
☒	☐	Increase transmissibility of a pathogen
☒	☐	Alter the host range of a pathogen
☒	☐	Enable evasion of diagnostic/detection modalities
☒	☐	Enable the weaponization of a biological agent or toxin
☒	☐	Any other potentially harmful combination of experiments and agents

ChIP-seq

Data deposition

☐ Confirm that both raw and final processed data have been deposited in a public database such as GEO.

☐ Confirm that you have deposited or provided access to graph files (e.g. BED files) for the called peaks.

Data access links<br>*May remain private before publication.*	NA
Files in database submission	NA
Genome browser session<br>(e.g. UCSC)	NA

Methodology

Replicates	NA
Sequencing depth	NA
Antibodies	NA
Peak calling parameters	NA
Data quality	NA
Software	NA

Flow Cytometry

Plots

Confirm that:

- [X] The axis labels state the marker and fluorochrome used (e.g. CD4-FITC).
- [x] The axis scales are clearly visible. Include numbers along axes only for bottom left plot of group (a 'group' is an analysis of identical markers).
- [X] All plots are contour plots with outliers or pseudocolor plots.
- [x] A numerical value for number of cells or percentage (with statistics) is provided.

Methodology

Sample preparation	For immunophenotypic analyses (performed on FACSCanto II; BD PharMingen), we used the antibodies listed in Table S2. Single stained and Fluorescence Minus One stained cells were used as controls. 7-AAD (Biolegend) was included in the sample preparation for flow cytometry according to the manufacturer's instructions to exclude dead cells from the analysis. Cell sorting was performed using MoFlo XDP Cell Sorter (Beckman Coulter) or FACS Aria Fusion (BD Biosciences). WBD protocol was performed according to 14. Briefly, after RBC lysis, the samples were incubated with mouse purified Rat anti-mouse CD16/CD32 (mouse BD Fc block™) solution (BD Bioscience) for 10 minutes at RT, to avoid unspecific binding to murine cells. After Fc blocking, cells were labeled with fluorescent antibodies against CD3, CD56, CD14, CD61/41, CD135, CD34, CD45RA (Biolegend) and CD33, CD66b, CD38, CD45, CD90, CD10, CD11c, CD19, CD7 and CD71 (BD Biosciences). After surface marking, the cells were incubated with PI (Biolegend) to stain dead cells. All samples were acquired through BD Symphony A5 (BD Bioscience) cytofluorimeter after Rainbow beads (Spherotech) calibration and raw data were collected through DIVA software (BD Biosciences). The data were subsequently analyzed with FlowJo software Version 10.5.3 (BD Biosciences) and the graphical output was generated through Prism 8.3.0 (GraphPad software). TSNE plots were generated through FlowJo software plug-in.
Instrument	MoFlo XDP Cell Sorter (Beckman Coulter), FACS Aria Fusion (BD Biosciences) and BD Symphony A5 (BD Bioscience)
Software	FlowJo software Version 10.5.3 (BD Biosciences) and the graphical output was generated through Prism 8.3.0 (GraphPad software). TSNE plots were generated through FlowJo software plug-in.
Cell population abundance	Cell populations abundances were calculated as relative percentage over the total cells analyzed by FACS. Absolute cell numbers were calculated by Burker chamber after trypan blue staining.
Gating strategy	In all Flow Cytometry procedures performed in this study, single cells were selected by representing FSC Height/Weight parameter vs Area. Then, cell populations were selected based on their FSC-A/SSC-A parameter.<br>Dead cells were excluded by Live/dead cell staining kit, that marked dead cells.<br>Cells expressing the mentioned markers were gated based on the respective negative controls of unstained cells, simple positive controls and SSC-A/FSC-A profiles to ensure the population.

- [x] Tick this box to confirm that a figure exemplifying the gating strategy is provided in the Supplementary Information.

Magnetic resonance imaging

Experimental design

Design type	NA
Design specifications	NA
Behavioral performance measures	NA

Acquisition

Imaging type(s)	NA
Field strength	NA
Sequence & imaging parameters	NA
Area of acquisition	NA

Diffusion MRI [ ] Used [x] Not used

Preprocessing

Preprocessing software	NA
Normalization	NA

Normalization template	NA
Noise and artifact removal	NA
Volume censoring	NA

Statistical modeling & inference

Model type and settings	NA
Effect(s) tested	NA

Specify type of analysis: ☐ Whole brain ☐ ROI-based ☐ Both

Statistic type for inference (See Eklund et al. 2016)	NA
Correction	NA

Models & analysis

n/a	Involved in the study
☒	☐ Functional and/or effective connectivity
☒	☐ Graph analysis
☒	☐ Multivariate modeling or predictive analysis
